# Acute Effects of a Session with The EXOPULSE Mollii Suit in a Fibromyalgia Patient: A Case Report

**DOI:** 10.3390/ijerph20032209

**Published:** 2023-01-26

**Authors:** Alejandro Rubio-Zarapuz, María Dolores Apolo-Arenas, Vicente Javier Clemente-Suárez, Ana Rodrigues Costa, David Pardo-Caballero, Jose A. Parraca

**Affiliations:** 1 Faculty of Sports Sciences, Universidad Europea de Madrid, Tajo Street, s/n, 28670 Madrid, Spain; 2 Facultad de Medicina y Ciencias de la Salud, Research Group FhysioH, Universidad de Extremadura, 06006 Badajoz, Spain; 3 Departamento de Ciências Médicas e da Saúde, Escola de Saúde e Desenvolvimento Humano, Universidade de Évora, 7004-516 Évora, Portugal; 4 AlgeaSalud, Clinica Neurorrehabilitación Deportiva, Avenida de Elvas, 06006 Badajoz, Spain; 5 Departamento de Desporto e Saúde, Escola de Saúde e Desenvolvimento Humano, Universidade de Évora, 7004-516 Évora, Portugal; 6 Comprehensive Health Research Centre (CHRC), University of Évora, 7004-516 Évora, Portugal

**Keywords:** fibromyalgia, electrostimulation, EXOPULSE mollii suit, pain, function, muscle oxygenation, autonomic modulation

## Abstract

Fibromyalgia is a chronic disorder characterized by widespread musculoskeletal pain and associated fatigue, sleep disturbances, and other cognitive and somatic symptoms. A multidisciplinary approach including pharmacological therapies along with behavioral therapy, exercise, patient education, and pain management is a possible solution for the treatment of this disease. The EXOPULSE Mollii^®^ method (EXONEURAL NETWORK AB, Danderyd, Sweden) is an innovative approach for non-invasive and self-administered electrical stimulation with multiple electrodes incorporated in a full-body suit, with already proven benefits for other diseases. Therefore, the present case report study aims to evaluate the effects that a 60 min session with the EXOPULSE Mollii suit has on a female fibromyalgia patient. After the intervention, we can conclude that a 60 min session with the EXOPULSE Mollii suit has beneficial effects on pain perception, muscle oxygenation, parasympathetic modulation, and function in a female fibromyalgia patient.

## 1. Introduction

Fibromyalgia is a chronic disorder characterized by widespread musculoskeletal pain and associated fatigue, sleep disturbances, and other cognitive and somatic symptoms [1], with a prevalence of 1.3–8% in the general population. The main cause of this disease is yet to be established. However, brain imaging studies and other research have uncovered evidence of altered signaling in neural pathways that transmit and receive pain in people with fibromyalgia, contributing to many of the problems that people with this disorder experience [2]. Further, fibromyalgia is a heterogeneous condition that is more common in females and is often associated with specific diseases such as infections, psychiatric or neurological disorders, diabetes, and rheumatic pathologies [3]. Along this line, current pharmacological treatments for patients suffering from this condition are mainly directed toward palliating symptoms, and relevant benefits are only achieved by a minority of patients, with a 50% pain reduction being achieved only by 10 to 25% of individuals [4]. Therefore, a multidisciplinary approach including pharmacological therapies, along with behavioral therapy, exercise, nutrition, patient education, and pain management, could be a solution for the treatment of this disease [5,6,7].

Further, the use of electrical stimulation with surface electrodes is a non-invasive therapeutic method used in patients’ central nervous system injuries, improving voluntary motor control and range of motion and reducing pain and spasticity [8]. Moreover, Scaturro et al. (2019) described the beneficial effects of a physical rehabilitation program composed by group exercise, laser, and the use of transcutaneous electrical nerve stimulation (TENS) treatment on fibromyalgia patients’ pain perception, fatigue, and overall life quality. Along this line, TENS is a safe intervention that delivers an electrical current through electrodes attached to the skin for pain control [9], activating central inhibitory pathways [10,11,12,13] and decreasing central excitability [11,14,15,16], with already proven effects in fibromyalgia patients for pain, fatigue, and hyperalgesia [17]. Furthermore, the EXOPULSE Mollii^®^ method (EXONEURAL NETWORK AB, Danderyd, Sweden) could be considered an evolution of the TENS system, as it is an innovative approach for non-invasive and self-administered electrical stimulation with multiple electrodes incorporated in a full-body suit [18]. This method is based on the concept of reciprocal inhibition elicited by stimulating the antagonist of a spastic muscle at low frequencies and low intensities by stimulating afferent nerve fibers of the antagonist muscle, which activates inhibitory Ia interneurons in the spinal cord and reduces the excitability of the agonist’s motor neuron [19,20]. Additionally, other mechanisms of action of the EXOPULSE Mollii suit may include neuroplastic changes in brain or spinal cord circuitries [21]. Further, the EXOPULSE Mollii suit has already proven beneficial in its use in children with cerebral palsy [22], spasticity [18], and chronic stroke patients [23]. Therefore, the present study aims to evaluate the acute effects that a 60 min session with the EXOPULSE Mollii suit has on a female fibromyalgia patient.

## 2. Materials and Methods

### 2.1. Study Design

The present study consists of a case report conducted to ascertain the acute effects of a treatment session with the EXOPULSE Mollii suit on a fibromyalgia patient.

### 2.2. Participant

A female fibromyalgia patient of 43 years of age and 1 year of diagnosis, weight of 85 kg, 164 cm height, and a 30.8 kg/cm^2^ Body Mass Index (BMI).

### 2.3. Intervention

**Figure 1:**
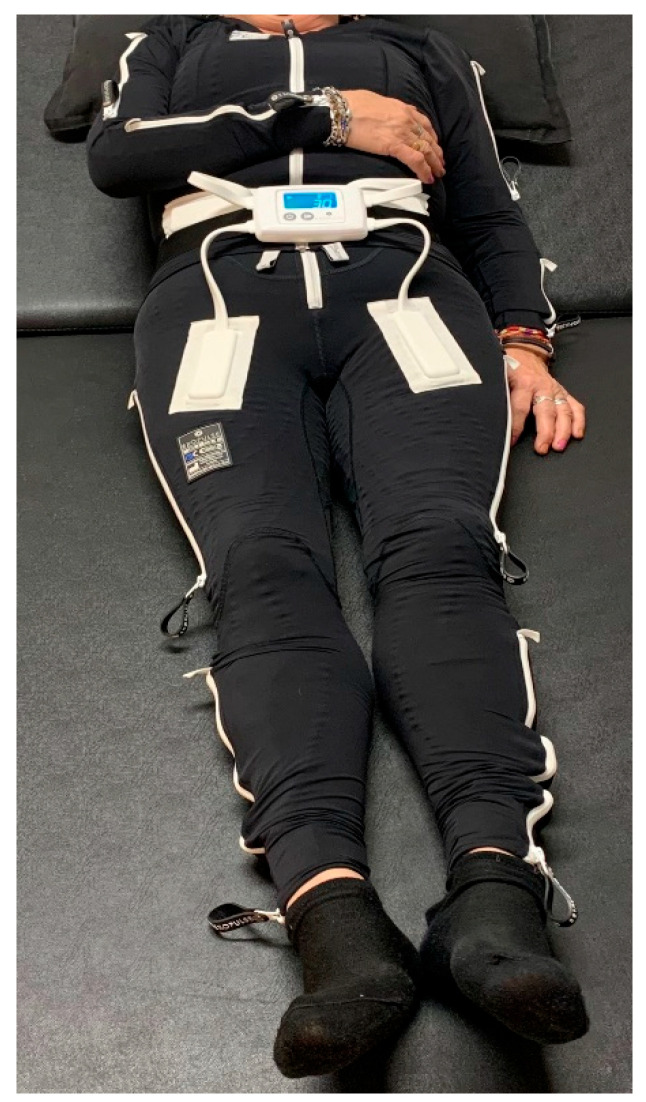
EXOPULSE Mollii Suit [30].

**Figure 2:**
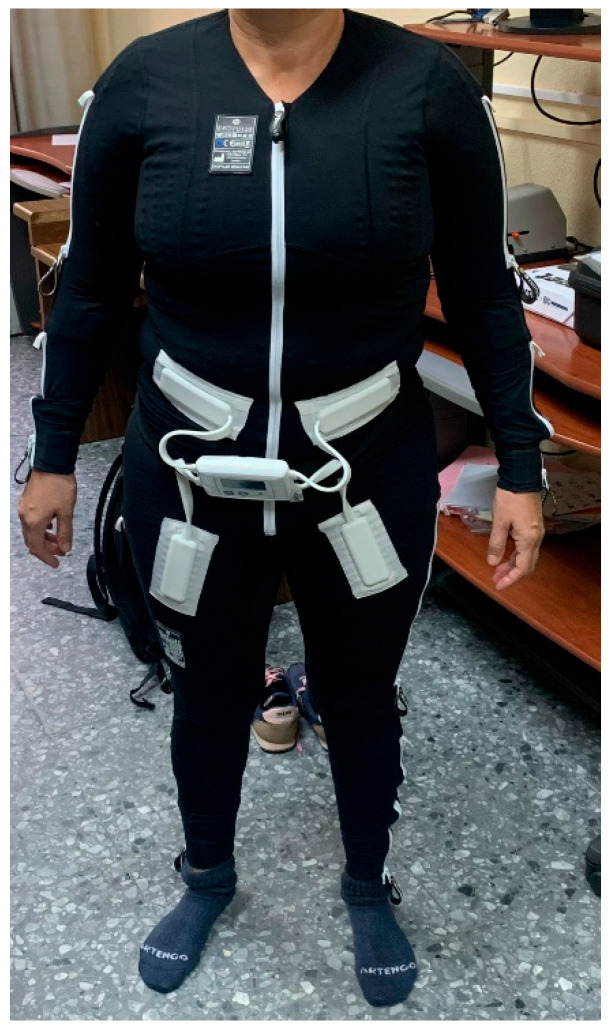
Mollii Control Unit [30].

The treatment with the EXOPULSE Mollii suit is described by the manufacturer as using 60 min sessions, with effects that vary between patients and that can last up to 48 h [24]. Therefore, to reach the aim of this study, the participant was subjected to a 60 min session with the EXOPULSE Mollii suit (Figure 1), with all 58 electrodes active with an intensity of 2 milliamperes (mA) and pulse width of 30 milliseconds (ms), as previously described in other studies using this same treatment [18,22,23,24,25,26,27,28,29]. Along this line, the intervention was carried out to examine the acute effects of the EXOPULSE Mollii suit in a fibromyalgia patient in the following manner. The participant arrived on site. The written consent was signed, and a basal evaluation was carried out. Then the participant suited up in the EXOPULSE Mollii suit with the help of a certified professional who ensured that all electrodes were correctly placed and contacted the patient’s skin. Once the suit was correctly placed, the control unit (Figure 2) was adhered to the suit, the patient was placed lying down and facing upwards on a massage table, the suit was turned on, and the session began. The patient was free to move throughout the session. Once the session was over, muscle oxygen levels and saliva recollection were carried out without taking off the suit. Afterwards, the patient changed clothes, and the rest of the post session evaluation was carried out.

### 2.4. Outcome Measures

All variables were evaluated prior to the intervention in basal conditions, and after the intervention.

#### 2.4.1. Thermography

The hot point was automatically assessed by a FLIR E8-XT system [31]. Two measures were taken of the back and front of the hands.

#### 2.4.2. Respiratory Variables

To measure the following variables, a spirometry test was conducted with a Vitalograph Asma1 spirometer [32]. The patient was asked to fully inhale (until the lungs were filled), close her lips around the mouthpiece, and exhale as quickly and forcefully as possible until the lungs were emptied, repeating this process 3 times [33]. The values of forced expiratory volume in 1 s (FEV1), 6 s (FEV6), and the ratio of both these values (FEV1/FEV6) [34] were registered. Further, chest perimeter difference between full air inspiration and full air expiration was also measured.

#### 2.4.3. Pain Severity

To assess pain severity, the Numeric Rating Scale (NRS) was used with a scale of 0–10, with 0 meaning “no pain”, and 10 meaning “the worst pain imaginable” [35].

#### 2.4.4. Muscle Oxygen Variables

Muscle oxygen saturation (SmO_2_), total hemoglobin (THb), deoxygenated hemoglobin (HHb), and oxygenated hemoglobin (O_2_Hb) values were measured using a portable NIRS sensor (Moxy, Fortiori Design LLC, Hutchinson, MN, USA) connected with the GoldenCheetah software (version 3.4, U.S.). This device, shown to be reliable at low and moderate intensity for the measuring of consumption of muscle oxygen (SmO_2_; ICC: r = 0.773–0.992) [36], was placed in the vast lateral quadriceps between the greater trochanter and the lateral femoral epicondyle. To reduce noise, a soft spline filter was applied using MATLAB^®^ software (The MathWorks, Inc., Natick, MA, USA). In the following, we used a second-order 6 Hz cut-off Butterworth filter, applied two times to the time series.

#### 2.4.5. Cortical Arousal

Cortical arousal was measured through the Critical Flicker Fusion Threshold (CFFT) in a viewing chamber (Lafayette Instrument Flicker Fusion Control Unit Model 12021), following the procedure conducted in previous studies [37,38].

#### 2.4.6. Functional Test

Adapted from the exercise test battery by Carbonell-Baeza et al. (2022) for fibromyalgia patients [39].

- Chair stand test: the number of times within 30 s that an individual can rise to a full stand from a seated position with back straight and feet flat on the floor, without pushing off with the arms.
- Handgrip strength test: measured using a grip dynamometer (Kuptone, model EH101). Each patient performs the test twice with the dominant hand, with the arm fully extended, forming a 30° angle in relation to the trunk.
- 10 m up and go test: consists of standing up from a chair and walking 10 m in the shortest time possible without running.
- One leg balance: the subject must keep balance on one leg with their eyes open as long as possible. This test is conducted on both legs.

#### 2.4.7. Pressure Pain Threshold

To obtain an estimate of the participant’s general pain sensitivity [40,41], pressure pain threshold (PPT) was measured in the right side of the participant’s body in two points previously assessed in other studies with fibromyalgia patients: the lateral epicondyle (2 cm distal to the epicondyles), and the inside of the knee (at the medial fat pad proximal to the joint line) [42,43]. The algometer was held perpendicular to the skin at the site, and subjects were instructed to verbally indicate when the pressure pain threshold was reached. The algometer used was a Wagner FPKTM algometer with a blunt rubber tip of 1 cm^2^.

#### 2.4.8. Salivary Biomarkers

Unstimulated whole saliva was collected at rest and after exercise for each participant by direct draining into an ice-cold collection tube (pre-weighted) for 3 min. Subjects refrained from eating and drinking for at least 1 h before collection. After saliva collection, tubes with the samples were weighted (for saliva flux evaluation, mL/min), centrifuged at 1500× *g* for 10 min to remove food and cell debris, and the supernatant was stored at −20 °C until analysis. Preceding analysis and saliva samples were thawed on ice and centrifuged for 30 min at 4 °C, 13,000× *g*, for removal of mucinous material [44]. Supernatant total protein concentration was assayed using the Bradford method [45].

### 2.5. Statistical Analysis

The statistical software SPSS (Statistical Package for Social Sciences, version 25) was used to perform the statistical analyses, with data being presented as mean ± standard deviation (SD).

### 2.6. Ethical Aspects

The current study followed the ethical standards recognized by the Declaration of Helsinki [46], the EEC Good Clinical Practice recommendations (document 111/3976/88, July 1990), and current Spanish legislation regulating clinical and biomedical research in humans, personal data protection, and bioethics (Royal Decree 561/1993 on clinical trials and 14/2007, 3rd July, for Biomedical research). The study was explained to the participant before starting, and the volunteer signed an informed consent form.

## 3. Results

Table 1 presents spirometry values before and after the intervention. FEV1 and FEV1/FEV6 values were higher prior to the intervention (pre:3.26 l, 0.84; post:3.11 l, 0.79), while FEV6 behaved the opposite way, with a higher value after the intervention (pre:3.86 l post:3.92 l). There were no differences in chest perimeter difference values before and after the intervention.

Muscle oxygen values are present in Table 2, with a clear increase in SmO_2_ and O_2_Hb after the intervention (pre: 41.8% ± 8.13, 4.79 ± 0.9; post: 52.2 ± 28.79, 5.97 ± 0.06), while HHb decreased after the intervention (pre: 6.68 ± 1.01; post: 5.47 ± 0.02), and there was no change in THb (pre: 11.47 ± 0.11; post: 11.44 ± 0.04).

Table 3 presents NRS values with a two-point decrease in pain after the intervention (pre: 7; post: 5), while PPT increased when measured in the epicondyle and decreased when measured in the knee (pre:1.4, 1.5; post: 1.7, 1.2).

Further on the Table 4, cortical arousal values before and after the intervention are very similar (pre: 36.1; post: 35.8), whereas saliva flux increased after the intervention (pre: 0.18; post: 0.42), and salivary proteins decreased after the intervention (pre: 5.587 ± 1.295; post: 3.73 ± 0.564).

Functional test results are present in Table 5, with the patient performing better in all tests after the intervention except for the monopodial balance with the left leg.

In Table 6, we can see temperature values of the hands and fingers of the patient measured with the thermographic camera, showing an overall decrease after the intervention in the temperature of all points measured.

## 4. Discussion

The present study is a novel case report study in the use of the EXOPULSE Mollii suit in a fibromyalgia patient procedure that has never been carried out in a scientific setting. As such, there is no previous research on this actual topic to compare our results with. Along this line, the main findings of our study are the drastic change in muscle oxygenation, with a 10.4% increase in SmO_2,_ and the changes in HHb and O_2_Hb distribution, with both values being more equal than before the intervention. These findings are in line with previous research on muscle oxygenation in fibromyalgia patients, which states that fibromyalgia patients have lower SmO_2_ and O_2_Hb and higher HHb compared to healthy controls [47]. Authors hypothesize that fibromyalgia patients have a mitochondrial dysfunction, making energy production insufficient due to an abnormal synthesis of adenosine-triphosphate (ATP) [48]. However, the introduction of a 60 min session with the EXOPULSE Mollii suit somewhat corrected this condition, increasing the basal value of SmO_2_ from 41.8% to 52.2% after the intervention, although it did not reach healthy population normal values of around 70–75% [49]. Further, NRS as a means for subjective pain perception decreased by two points after the intervention, changing from seven to five points. These findings could also be related with mitochondrial disfunction, as pain has already been related to this alteration [50], and as this mitochondrial dysfunction is corrected by the intervention as shown by an increase in muscle oxygenation. ATP production may be corrected, and thus, muscle oxygen demands decrease, decreasing the patient’s pain perception, and increasing the patient’s performance in functional tests as shown by our results.

Along this line, the patient performed better in all tests after the intervention, which could be attributed to this increase in muscle oxygenation, or to a better ATP production and the reduction in pain allowing the patient to better exert herself in the tests. Further, we should highlight the increase in balance with the right leg, where the subject went from holding only for 3 s to being able to maintain balance for 30 s before having to stop. As previously stated, this increase in performance could be attributed to the reduction of pain, or to the better oxygen usage acquired after the intervention. However, further research should be conducted on the duration of these effects, and whether these effects could be made chronic with successive sessions.

Furthermore, it has been demonstrated that fibromyalgia patients have a higher sympathetic nervous system activation in basal conditions than healthy individuals, which is consistent with a decrease in salivary flow [51,52]. Along this line, after the intervention, an increase in salivary flow is observed, without a significant change in protein concentration, contrary to the decrease in flow produced by a fatigue protocol implemented by Costa et al. 2022 [52], which was correlated with an increase in sympathetic nervous system activation. This increase in flow after the intervention with the suit suggests an increase in parasympathetic tonus. The decrease of temperature observed in the hands of the patient could also be related to a higher parasympathetic nervous system activation, as there, blood flow reduction occurs with parasympathetic activation [53]. Further, a reduction in sympathetic activation could also be consistent with the reduction in the patient pain perception.

To summarize, in this study, we have found significant changes in muscle oxygenation, functional test performance, and subjective pain perception measured through the NRS, saliva flux, and hand temperature, which in turn suggest a better mitochondrial function and an increase in parasympathetic tone. Along this line, now that a successful case report has been produced, it would be of great interest to implement this same procedure with a wider sample. Moreover, the duration of these acute effects on this fibromyalgia patient should be studied, and the effects of successive sessions should also be studied.

## 5. Conclusions

We can conclude that a 60 min session with the EXOPULSE Mollii suit has beneficial effects on pain perception, muscle oxygenation, parasympathetic modulation, and function in a female fibromyalgia patient.

## Figures and Tables

**Table 1:** Respiratory values before and after the intervention.

Variables	Pre	Post
FEV1, L	3.26	3.11
FEV6, L	3.86	3.92
FEV1/FEV6	0.84	0.79
Chest perimeter difference, cm	8	8

FEV1: forced expiratory volume in 1 s. FEV6: forced expiratory volume in 6 s.

**Table 2:** Muscle oxygen values before and after the intervention.

Variables	Pre	Post
SmO_2_, %	41.8 ± 8.13	52.2 ± 28.79
THb, g/dL	11.47 ± 0.11	11.44 ± 0.04
HHb, g/dL	6.68 ± 1.01	5.47 ± 0.02
O_2_Hb, g/dL	4.79 ± 0.9	5.97 ± 0.06

Data are presented as mean ± standard deviation. SmO_2_: muscle oxygen saturation. THb: total hemoglobin. HHb: deoxygenated hemoglobin. O_2_Hb: oxygenated hemoglobin.

**Table 3:** NRS and PPT values before and after the intervention.

Variables	Pre	Post
NRS, 0–10	7	5
PPT epicondyle, kg	1.4	1.7
PPT knee, kg	1.5	1.2

NRS: Numeric Rating Scale. PPT: Pressure pain threshold.

**Table 4:** Cortical arousal and saliva values before and after the intervention.

Variables	Pre	Post
Cortical Arousal, Hz	36.1	35.8
Saliva flux, mL/min	0.18	0.42
Salivary Proteins, mg/mL	5.587 ± 1.295	3.73 ± 0.564

Data are presented as mean ± standard deviation for salivary protein values.

**Table 5:** Functional test values before and after the intervention.

Variables	Pre	Post
Chair stand test, n	5	8
Handgrip strength test, kg	15.9	17.5
10 m up and go test, s	6.83	6.02
One leg balance right, s	3.04	30

**Table 6:** Temperature values of the hands and fingers of the patient.

Part of the Hand	Side	End	Pre	Post
Palm	Right		33.4	30.3
	Left		33.1	31.0
Thumb	Right	Proximal	32.8	28.4
		Distal	32.1	26.4
	Left	Proximal	31.8	28.7
		Distal	31.1	27.2
Index finger	Right	Proximal	32.7	27.3
		Distal	32.2	26.4
	Left	Proximal	30.3	27.9
		Distal	29.2	27.2
Middle finger	Right	Proximal	31.5	27.2
		Distal	30.9	26.3
	Left	Proximal	30.5	28.2
		Distal	29.7	27.4
Ring finger	Right	Proximal	30.4	26.7
		Distal	30.6	26.2
	Left	Proximal	30.8	28.2
		Distal	29.4	27.1
Little finger	Right	Proximal	29.1	26.6
		Distal	28.5	25.7
	Left	Proximal	30.6	27.7
		Distal	29.1	27.0

## Data Availability

Not applicable.
